# Coronary artery anomalies: A case of the “malignant” left coronary artery and its surgical management

**DOI:** 10.1515/med-2025-1298

**Published:** 2025-10-18

**Authors:** Waliya Badar Hossain, Kumail Abbas Khan, Sophia Khattak, Farhan Shahid, Sohail Q. Khan

**Affiliations:** Institute of Cardiovascular Sciences, University of Birmingham, Birmingham, United Kingdom; The Kidney Centre Post Graduate Training Institute, Karachi, Pakistan; Department of Interventional Cardiology, Queen Elizabeth Hospital, Birmingham, United Kingdom; Armed Forces Institute of Cardiology, Rawalpindi, Pakistan; Institute of Cardiovascular Sciences, University of Birmingham, Birmingham, United Kingdom

**Keywords:** case report, anomalous left coronary artery, inter-arterial/malignant variant, ischemia, coronary artery bypass grafting, sudden cardiac death

## Abstract

**Background:**

Any variance of the normal coronary vasculature is regarded as a coronary artery anomaly (CAA). An atypical left coronary artery arising from the right aortic sinus of Valsalva has been identified as the rarest CAA and the second leading cause of sudden cardiac death (SCD) in young people. Prompt identification of the anomalous vessel is essential to mitigate early mortality risk.

**Case summary:**

A 33-year-old male, ex-smoker presented with exertional breathlessness and retrosternal chest tightness for the past 1 year. Past history also revealed a significant decline in his exercise tolerance. His initial work-up, which included a resting electrocardiogram and echocardiogram were within normal limits. His exercise tolerance test however, was discontinued early due to the onset of symptoms, and his exercise stress echocardiogram proved positive for angina at a low workload. The cardiac computed tomography angiography revealed a common origin for the left and right coronary systems from the right ostium, with the left main stem following an inter-arterial course and showing mild, diffuse narrowing. The patient was green-lit for coronary artery bypass grafting (CABG). Post recovery and almost 2 years on, he has remained symptom-free and has regained his physical activity.

**Conclusion:**

This case underlines the need for prompt identification and subsequent management of anomalous coronary arteries, given their association with SCD. Timely surgical intervention such as CABG can greatly mitigate the risk of grievous complications. The patient’s complication-free postop recovery and resumption of physical activity, assures the reader that it is a viable and durable option with long-term quality of life improvement.

## 1 Introduction

A coronary artery anomaly (CAA) is defined as any deviation from normal coronary vasculature, encountered in less than 1% of the population [1]. General consensus places their global incidence to be around 0.3 and 5.6% [2], but exact numbers cannot be accurately determined due to a lack of routine cardiovascular screening [3]. One such anomaly is the atypical left coronary artery (LCA) arising from the right aortic sinus of Valsalva (RASV). Widely considered to be the rarest CAA, it accounts for only 0.03% of overall cases [4]. While many CAAs remain asymptomatic throughout life, this particular variant, can cause sudden cardiac death (SCD) when activated by vigorous exercise [5]. Thus, it is often referred to as the “malignant variant,” and is widely recognised as the second most common cause of SCD in individuals less than 35 years of age [3]. Given the fatality of this anomaly, time sensitive identification and surgical revascularisation is required in order to mitigate the risk of mortality [6].

Here, we present a case of an anomalous LCA that was managed with coronary artery bypass grafting (CABG). While CABG is usually not considered as a first line therapy for anomalous origin of coronary artery from opposite sinus in young patients, this case highlights its effectiveness as a key vascularisation strategy in select diegeses.

## 2 Case presentation

**Figure 1:**
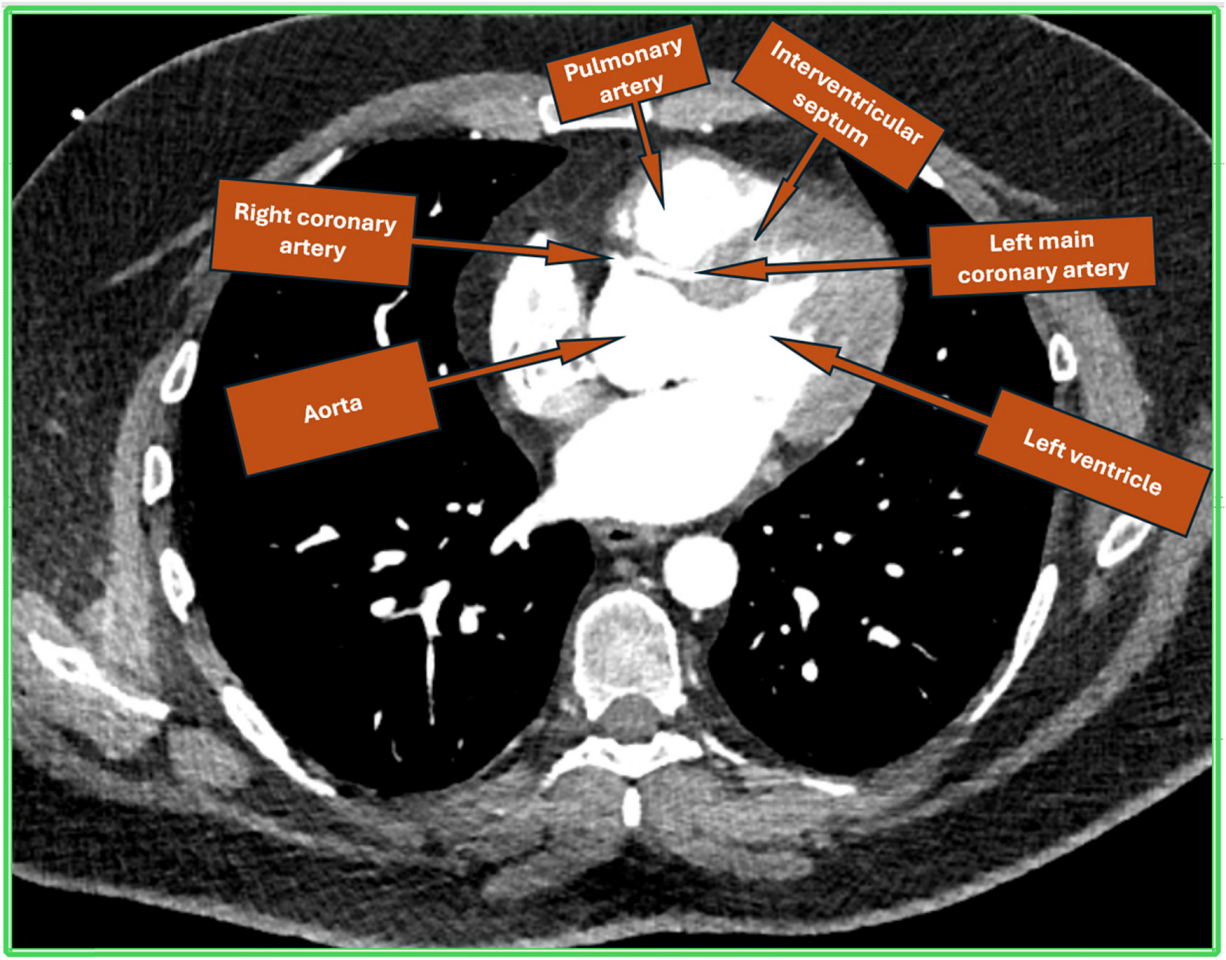
Cardiac computed tomogram showing single coronary origin from right coronary cusp and LCA having inter-arterial course between aorta and PA followed by course in the interventricular septum.

**Figure 2:**
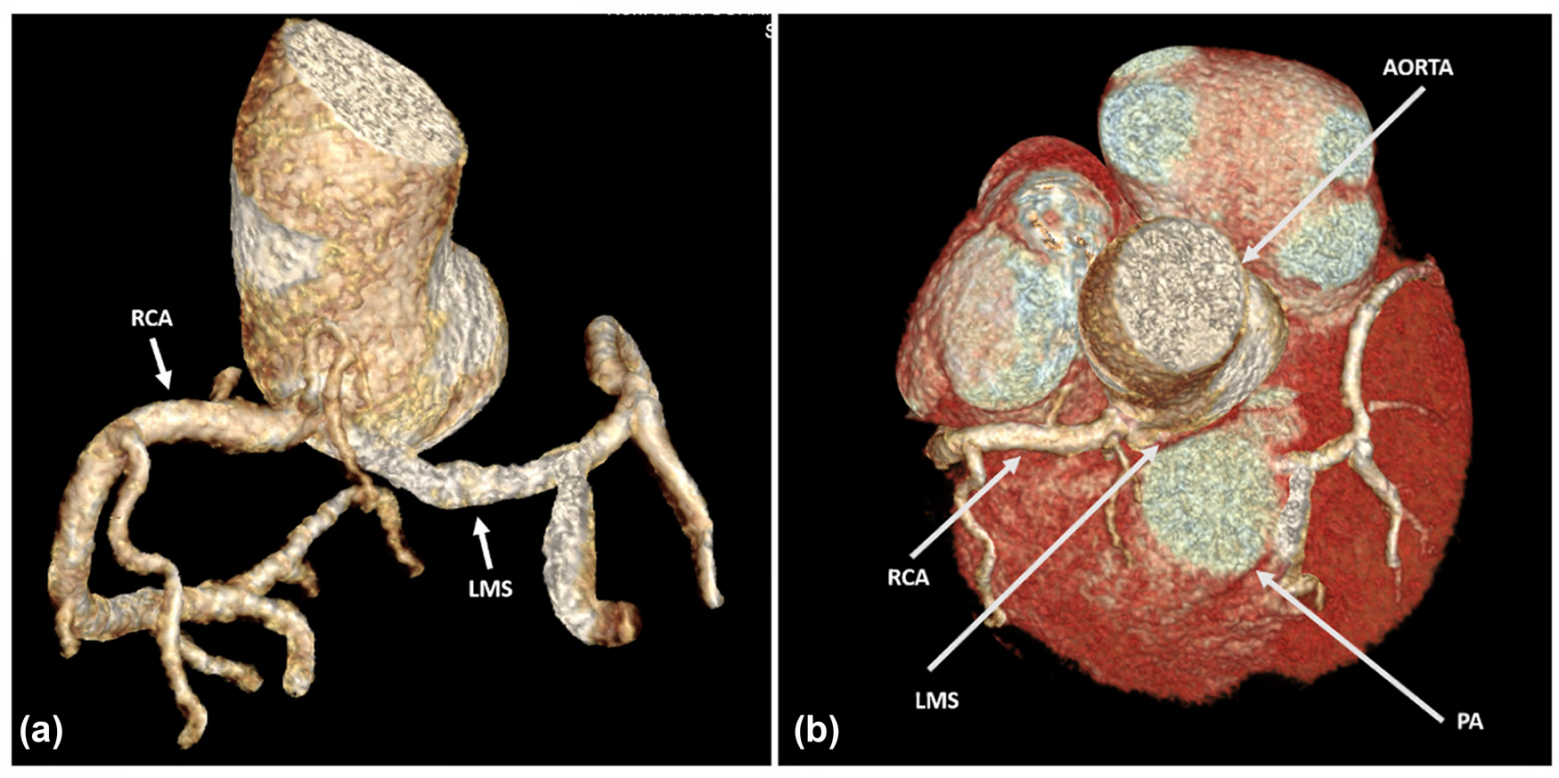
(a) Anterior view of a 3D rendered image of the CT coronary angiogram demonstrating anomalous origin of the LMS and right coronary artery (RCA) arising from the common origin of the right coronary cusp. (b) Aerial view depicting the LMS and RCA sharing a common origin right coronary cusp. The LMS can be seen to narrow as it traverses the inter-arterial between the aorta and PA.

A 33-year-old male with a history of high BMI and smoking presented to the Rapid Access Chest Pain Clinic with exertional breathlessness and retrosternal chest tightness for the past 1 year. He reported a sharp decline in his exercise tolerance with recent difficulty when walking as little as 300 yards. One year earlier, he was able to exercise at high intensity in the gym. His workup included coronary computed tomographic angiography (CCTA) which demonstrated a common origin for the left and right coronary systems, via the right ostium of Valsalva (Figure 1). The left main stem (LMS), after taking off anomalously from the right coronary cusp, adopted an inter-arterial course between the main pulmonary trunk and aorta (Figure 2), progressing as a long intramural course through the aortic wall, before opening out into the aortic lumen. Throughout its path, the vessel exhibited mild, diffuse stenosis, likely due a combination of the slit-like ostial opening and dynamic compression between the aorta and pulmonary artery (PA). In comparison, the right coronary artery appeared to be dominant and of a larger calibre. No significant atheroma or stenosis could be visualised in either the main coronary system or in any important side branches.

A transthoracic echo revealed normal biventricular function with ejection fraction of 60%, with no valvular dysfunction. However, both cardiac stress tests had to be terminated early, due to the development of symptoms. His BRUCE protocol treadmill test was aborted after only six and a half minutes due to onset of chest tightness although no arrhythmias or ST changes were detected throughout its duration. And the stress echocardiogram detected the presence of angina at a low workload. The 24 h Holter monitoring displayed sinus rhythm throughout with the presence of three unifocal isolated ventricular ectopic beats. After extensive multidisciplinary deliberation keeping the primary focus on his significant, progressive and limiting anginal symptoms, he was referred for elective CABG of the left coronary system. During procedure, the native anomalous LCA was ligated proximally to bypass native flow, followed by anastomosis of the mid left anterior descending (LAD) and obtuse marginal 1 (OM1) arteries with the left internal mammary artery (LIMA) and saphenous vein grafts, respectively. The post-operative course was uneventful and the patient was discharged from hospital after 6 days.

Two years post-procedure, he remains symptom free with full restoration of previous baseline physical activity.


**Informed consent:** Written informed consent was obtained prior to the publication of this case report and the accompanying images.

## 3 Discussion

CAAs have gained considerable traction in recent decades; previously regarded as benign incidental findings, their bearing on SCD is far too significant to be ignored [6]. The ectopic left coronary system identified within this case, is widely considered as the “malignant” variant, which traverses between the aorta and the PA, it is not only regarded as the rarest form of anomalous LCA [7], but also as the name suggests, the most dangerous [8,9].

**Figure 3:**
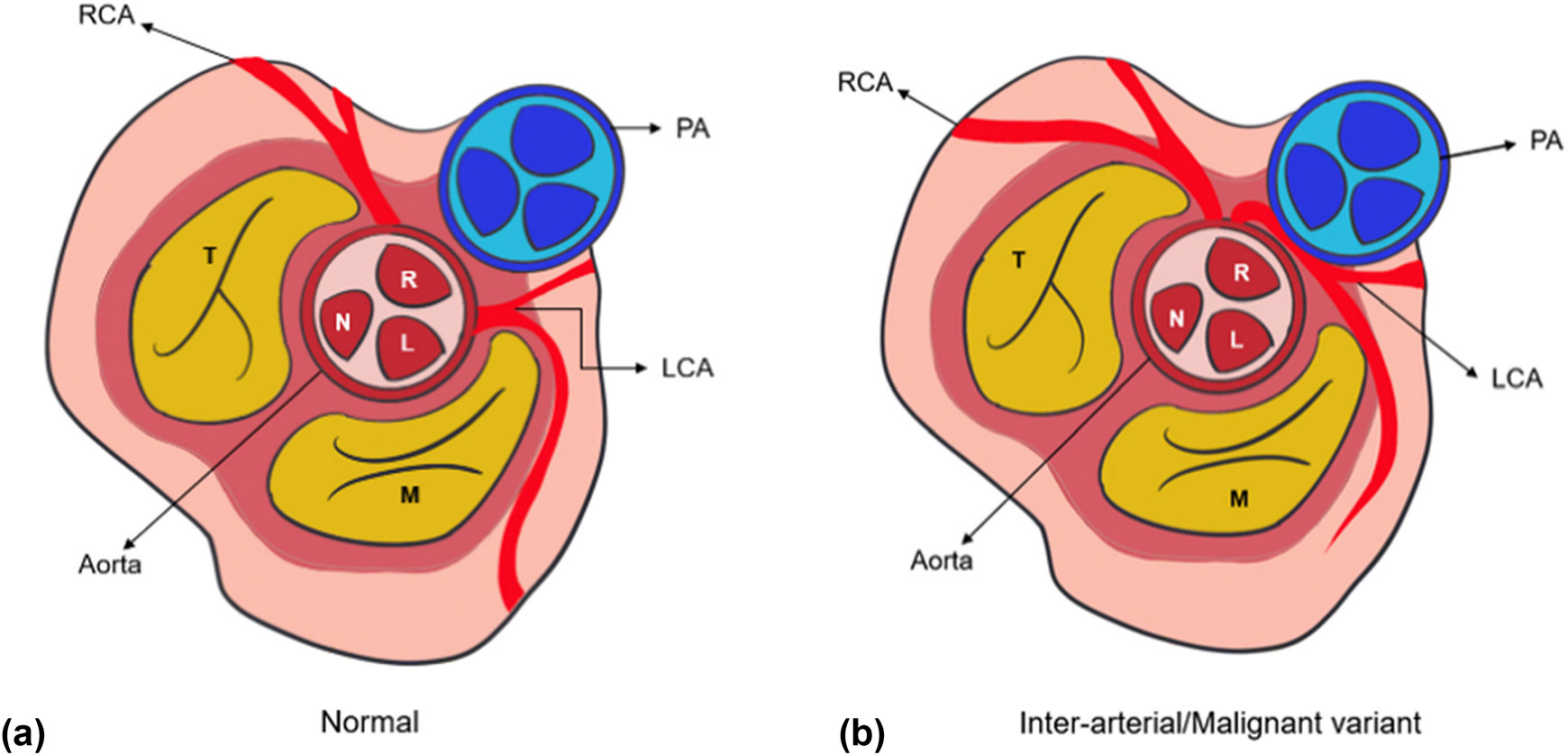
(a) Schematic representation of normal coronary anatomy, with LCA arising from left aortic sinus of Valsalva. (b) Schematic representation of anomalous origin of LCA arising from RASV. RCA: right coronary artery; PA: pulmonary artery; LCA: left coronary artery; R: right sinus of Valsalva; L: left sinus of Valsalva; N: non-coronary sinus; M: mitral valve, T: tricuspid valve.

There are several theories that have been developed over the years to explain the mechanism behind the “malignancy” of this variant. The oldest and most widely acknowledged, is the distention of the great vessels, i.e., the aorta and the PA during vigorous exertion. Their expansion within the limited sulcal space, compresses the anomalous LCA as it courses between them, leading to a significant compromise of blood flow [7]. However, this theory has largely been debunked due to the inexplicability of a low-pressure PA generating enough internal force to counteract the high-flow system of the LCA [10]. Another hypothesis is based on the presence of high-risk anatomical features that can lead to intermittent vessel occlusion. One offender being the slit-shaped right ostial opening formed because of the high take-off angle adopted by the exiting vessel, the other, the kinking of the anomalous LCA due to aortic expansion during systole. Combined, these two can lead to episodic myocardial ischemia which potentiates a lethal arrhythmia and the possibility of sudden death during exertion [11] which is unheard of when the right and left coronary arteries follow their standard origin and course (Figure 3a).

**Figure 4:**
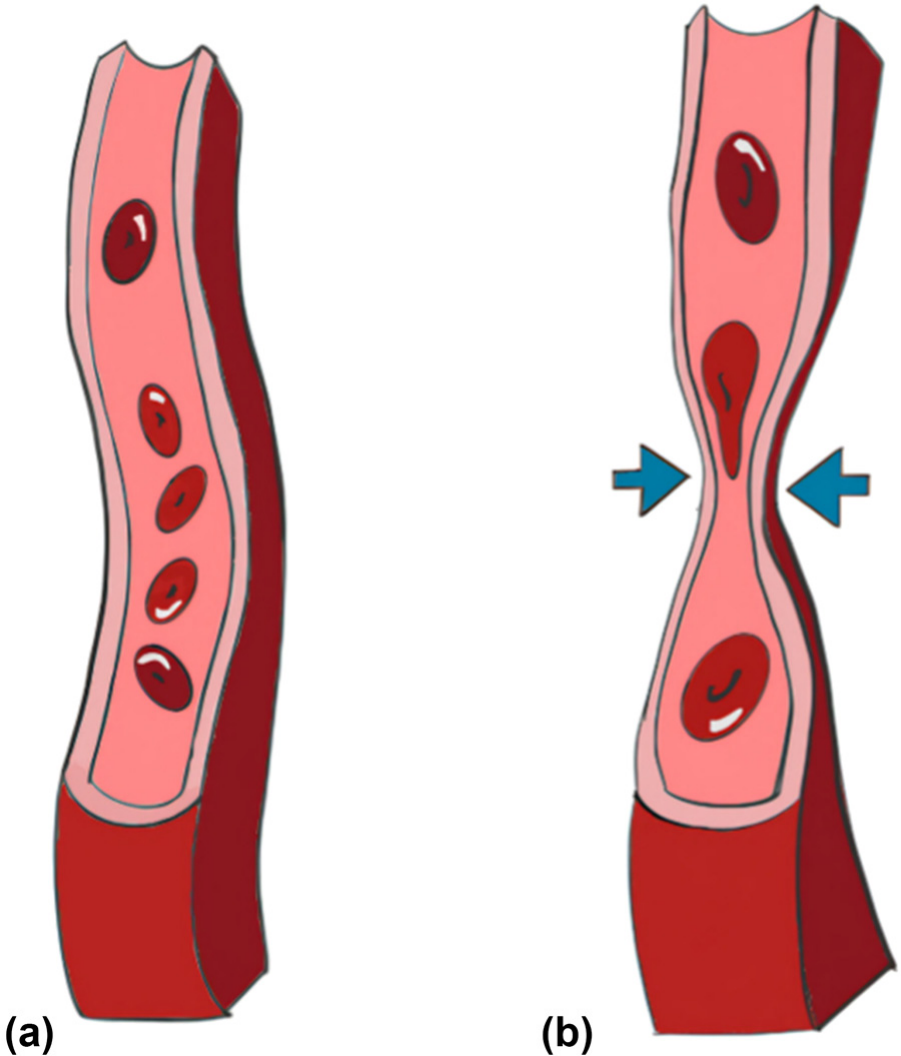
(a) Schematic representation of normal LCA with a wide and unobstructed lumen. (b) Schematic representation of the inter-arterial LCA, being compressed by the major arteries.

An alternative theory and arguably the most likely scenario indicates the presence of an intramural segment. Before the LMS exits the aorta to enter the sulcus, its initial segment is embedded within the aortic walls. These two vessels share a common tunica media, with no dividing adventitia in between. This leaves the comparatively thinner LCA susceptible to compression by the thick, elastic fibres of the aortic medial wall during vigorous contractile activity [12], presented schematically in Figure 3b. The more vigorous the exertion, the faster the rate of contraction, the greater the impediment to blood flow and the greater likelihood of developing a sudden cardiac arrest (Figure 4).

Furthermore, in this particular case, the LAD and the obtuse marginal coming off the LMS were found to have significantly reduced diameters than the known standard. Both vessel diameters were measured during the surgery and found to be 1.25 mm each, respectively. Previous studies in which the coronary vessels were examined and subsequently measured have determined that the narrower the calibre of the vessel, the greater the risk of developing a more severe form of coronary artery disease (CAD) [13]. Thus, this inversely proportional relationship would have only added to the vascular dysfunction and thereby worsened the burden of disease.

As previously mentioned, this ACAOS variant is exceptionally rare with precise incidence figures remaining elusive due to the absence of routine cardiovascular screening. This is evidenced in the largest angiographic study to date which screened 126,595 participants for the presence of CAAs, identifying 1,686 cases and establishing an incidence of 1.3%. Of these diminutive numbers, only 0.017% of the surveyed population possessed an anomalous LAD from their RASV [14]. Nevertheless, no other CAA in history has been more strongly associated with SCD than the anomalous LCA from the opposite sinus (ALCAOS) [15,16]. When combined with a congenitally narrow-calibre vessel, this can only exacerbate hemodynamic instability, increasing the risk of a myocardial infarction.

Their near-negligible presence thus proves a challenge towards establishing evidence-based guidelines on the proper diagnostic and therapeutic management. Practitioners will often proceed based on local knowledge, surgical expertise, institutional preference and most importantly, the individual vascular anatomy. More often than not, the decision involves choosing between a less invasive and a more invasive approach specifically, between CABG or unroofing. However, each technique carries its own set of drawbacks.

CABG is regarded as the revascularisation technique of choice for left CAD, under whose umbrella, left ACA’s take shelter [17]. It is associated with later survival and a reduced risk of major, inimical cerebrovascular and cardiac events [18]. Meanwhile, surgical unroofing is regarded as the surgical intervention of choice for managing the anomalous origin of the left coronary artery (AAOLCA) [19]. However, in our case, unroofing presented with its own share of technical difficulties due to the presence of a long intramural segment in the proximal course of the anomalous vessel. Anatomical features such as these can hinder the unroofing process, leading to an incomplete attempt during the procedure. This, in turn increases the risk of resistant post-procedural narrowing, potentially necessitating surgical re-evaluation [4]. Studies have shown that patients undergoing unroofing may require re-intervention further down the line due to inadequacies in either flow restoration or stenosis relief complications that can lead to new onset aortic regurgitation or rebound ischemia [17]. In some cases, even calling for a rescue CABG due to primary failure [4].

In the adult population, CABG is considered a low-risk and viable solution for malignant CAAs, especially since it is a commonly performed procedure, and adult cardiac surgeons are highly proficient in executing it [20]. This approach can often reduce technical challenges such as restenosis and flow reduction later on.

One of the main concerns regarding CABG for AAOLCA, is that of native competitive flow within the anomalous vessel leading to early graft failure [6]. In order to mitigate this, the native left coronary in our patient was surgically ligated, followed by end-to-side anastomosis of the mid-LAD to the LIMA graft. Not only does this circumvent the issue of native-vessel competitive flow but also maximises long-term viability of the graft [21]. In fact, the use of the LIMA graft has been shown to possess a patency rate surpassing 90% at 15 years [20].

Additionally, in the presence of documented ischaemia, apparent in our case, CABG has proven to show good early and midterm results, taking precedence over other proposed methods [22]. This rationale is further strengthened by the European Society of Cardiology guidelines, which classify a symptomatic patient as a Class IC indication for surgery, particularly in the presence of high-risk anatomy [23].

While most guidelines advocate for unroofing as the primary surgical approach, with CABG often reserved as a rescue procedure for thrombosed or diseased vessels, individual cases like ours, highlight unique opportunities to explore a tailored approach. Furthermore, surgical unroofing as a primary recommendation for treating AAOLCA has predominantly stemmed from paediatric cohorts, CABG provides an alternative albeit equally persuasive approach in an adult patient, especially given the complex vascular anatomy.

Faced with a lack of robust, long-term outcome data, surgical decision making must remain flexible. The multidisciplinary team must evaluate their patient’s vascular anatomy, physiology, and other factors in order to craft and execute the ideal corrective strategy.

## 4 Conclusion

In conclusion, reported cases of the anomalous LCA are too few and far between to establish a one-size-fits-all treatment approach. Each individual case provides an opportunity to tailor management according to individual anatomical and clinical presentation. In our case, CABG was considered as a viable treatment option, based on the following criterion: imaging findings, intraoperative discovery, and institutional facility. Multidisciplinary discussions among cardiac surgeons, interventional cardiologists, and cardiac imaging specialists were paramount when it came to weighing the various treatment strategies and ensuring the best outcome for the patient. This fortifies the critical role that collaborative decision-making plays when confronted with these uniquely complex anomalies.
